# Supplementary material for: Content-rich biological network constructed by mining PubMed abstracts
Source: BMC Bioinformatics. 2004 Oct 8;5:147. doi: 10.1186/1471-2105-5-147 (PMC528731; doi:10.1186/1471-2105-5-147)
Supplement: Additional File 2 — The original results of the above study (non-essential files are deleted to keep the file size under the limit set by BMC bioinformatics). [file 1471-2105-5-147-S2.bz2 › chilibotAdditionalFile2/dip50/363ID8663044E5753/html/FGF1_FGFR2.html]

 


 **FGF1** and **FGFR2** 
  
Found 1103 abstracts in PubMed, retrieved 50.  
 

 What does Google say? 
 PDF only 
| .edu only 

---

**Interactive relationship** (e.g. stimulation, inhibition, etc)

**Neutral relationship**- These findings suggest that FGFR1 and  **FGFR2**  in myofibroblasts may be responsible for the signal transduction of FGF  [ **FGF1** ]  during the wound healing process.  Ref: 12648559 Arch Oral Biol, 2003
- Keratinocyte growth factor receptor  [ **FGFR2** ]  KGFR  [ **FGFR2** ] , also known as fibroblast growth factor receptor FGFR 2  [ **FGFR2** ]  IIIb, is located in many types of epithelial cells and is activated by four known ligands FGF 1  [ **FGF1** ] , FGF  [ **FGF1** ]  3, FGF  [ **FGF1** ]  7 also known as KGF and FGF  [ **FGF1** ]  10 that are predominantly synthesized by mesenchymal cells.  Ref: 12608893 Pathol Int, 2003
- The relative mitogenic efficacy of basic FGF  [ **FGF1** ] , acidic FGF  [ **FGF1** ] , or KGF was in correlation with their affinities for the KGF receptor KGFR  [ **FGFR2** ] .  Ref: 12761878 J Cell Biochem, 2003

**Non-interactive relationship** (e.g. studied together, co-existance, homology, etc.)

- Previously, FGF  [ **FGF1** ]  7 mRNA expression was reported in VSMC, but KGFR  [ **FGFR2** ]  mRNA was not detected.  Ref: 12608893 Pathol Int, 2003
- Since the presence of FGF  [ **FGF1** ]  receptors FGFRs determines cellular responsiveness, we examined the localisation of FGFR1,  **FGFR2**  and FGFR3 expression by immunohistochemistry throughout the repair of full thickness excisional wounds up to 28 days after wounding.  Ref: 12648559 Arch Oral Biol, 2003
- FGF10 and FGF  [ **FGF1** ]  receptor FGFR 2  [ **FGFR2** ]  immunostaining showed a similar epithelial expression pattern.  Ref: 12697038 J Endocrinol, 2003
- Using semiquantitative reverse transcription polymerase chain reaction, we examined the expression of insulin like growth factor 1 Igf 1, fibroblast growth factor 2 Fgf  [ **FGF1** ]  2, and their receptors Igf 1r, Fgfr1,  **Fgfr2** , and Fgfr3 in the MCC of 28 day old rats at, and 14 days after placing intraoral appliances designed to produce a lateral functional shift of the mandible.  Ref: 12594422 Am J Orthod Dentofacial Orthop, 2003
- Expression of the FGF  [ **FGF1** ]  receptor 2 gene  **fgfr2**  during embryogenesis in the zebrafish Danio rerio.  Ref: 12617798 Gene Expr Patterns, 2002
- This provides an initial description of the expression in the developing skull of the genes encoding the secreted molecules BMP 2, BMP 4, BMP 7, the transmembrane FGF  [ **FGF1** ]  receptors FGFR 1, FGFR 2  [ **FGFR2** ] , FGFR 4, the transcription factors Msx1, Msx2, and Twist, as well as Goosecoid and the early membranous bone differentiation marker osteopontin.  Ref: 12710966 Dev Biol, 2003
